# A Game Changing, One of Its Kind Flap: The Boomerang-Shaped Extended Rectus Abdominis Myocutaneous Flap with Latissimus Dorsi Myocutaneous Flap

**DOI:** 10.1055/s-0045-1808096

**Published:** 2025-08-08

**Authors:** Srikanth Vasudevan, Ananteshwar Y. N., Mayur Shetty, Annika Marwah, Pooja Shetty, Serena B., Aashita Yande, Chinmay Tewari

**Affiliations:** 1 Department of Plastic and Reconstructive Surgery, Manipal Hospital, Bangalore, Karnataka, India

**Keywords:** soft tissue reconstruction of extremities, longest free flap, boomerang-shaped extended rectus abdominis myocutaneous flap

## Abstract

Reconstruction of extensive limb defects is particularly difficult when standard flaps like the anterolateral thigh flap, latissimus dorsi (LD) flap, or the less popular large flap, boomerang-shaped extended rectus abdominis (BERAM) flap, are insufficient to cover the extensiveness of the raw area, even after extending with vein grafts. The BERAM and LD myocutaneous flap combines two flap areas into a single tissue unit, offering a novel solution to the issue. Interposition vein grafts help extending the reach further and avoids the anastomosis in a probable zone of injury area. In our experience, it gives the largest possible tissue for coverage compared to any two separate free flaps especially in children with a good caliber, reliable pedicle.

## Introduction

Limb salvage has always been the goal of all reconstructive surgeons and complex limb defects challenge surgeons to come up with innovative modifications.

Boomerang-shaped extended rectus abdominis (BERAM) and latissimus dorsi (LD) flaps individually are well documented for reconstructive procedures. 1 2 Vein grafts have been used to extend the reach of microvascular flaps. 3

Extensive leg defects that require flap cover longer than that provided by commonly done free flaps like the anterolateral thigh (ALT) and LD flap even in chimeric forms is a challenging problem. When vascularity of foot and sole sensation is intact, limb salvage attempt is warranted. 4

A combination of all, which has not been described before, were used to perform a microvascular free tissue transfer to salvage the lower limb of a young boy post a devastating degloving injury.

## Case Report

A 16-year-old boy with an alleged history of road traffic accident presented to us 3 days after the injury with a request to salvage the limb as he was counseled for above-knee amputation where he was resuscitated and stabilized with an external fixator. We were presented with multiple challenges; delayed presentation with a circumferential degloving injury of the right lower limb of 53 * 24 cm extending from the upper thigh to the lower third of the leg with exposed knee joint, with only the medial gastrocnemius and part of soleus intact, full length of fractured tibia exposed and absent fibula and a single vessel limb with only the posterior tibial (PT) vessels and nerve present ( Supplementary Fig. 1 , available in the online version).

Intact vascularity and sole sensations along with age prompted us toward limb salvage after thorough counseling. Options were a double free flap, which on planning in reverse was still falling short because of the circumferential exposure of the knee joint and the length of the defect. An extended BERAM flap with LD myocutaneous flap as one continuous tissue unit emerged as an option, which on planning gave us the maximum possible tissue coverage. Still, the reach of the pedicle was short so we planned to extend it by the use of vein grafts. Marking was done in reverse with maximum width permissible for primary closure.

**Fig. 1:**
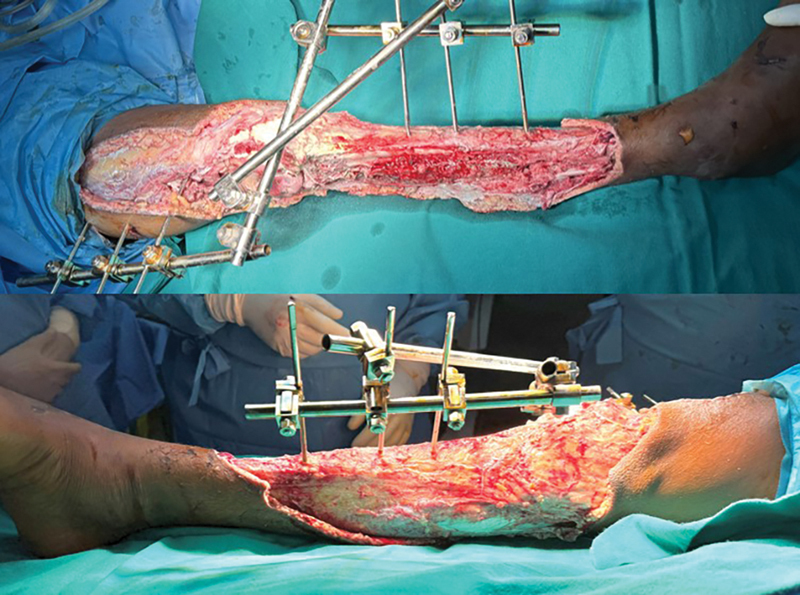
Right leg defect post-debridement.

**Fig. 2:**
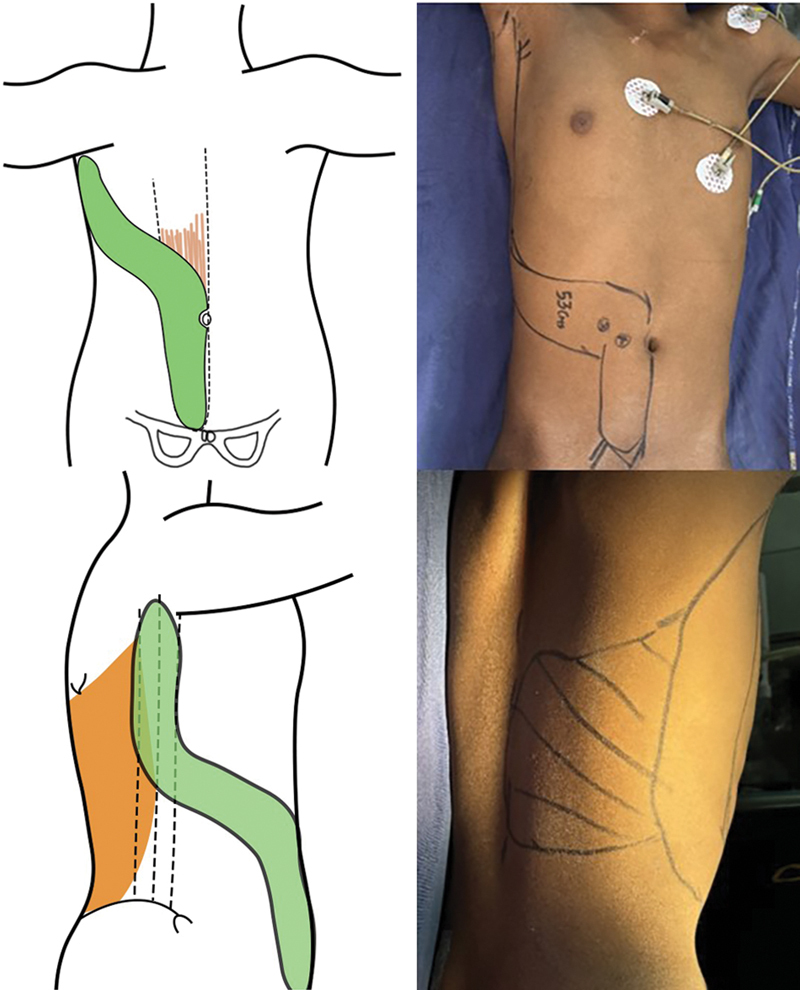
Plan: Boomerang-shaped extended rectus abdominis myocutaneous flap with latissimus dorsi myocutaneous flap.

**Fig. 3:**
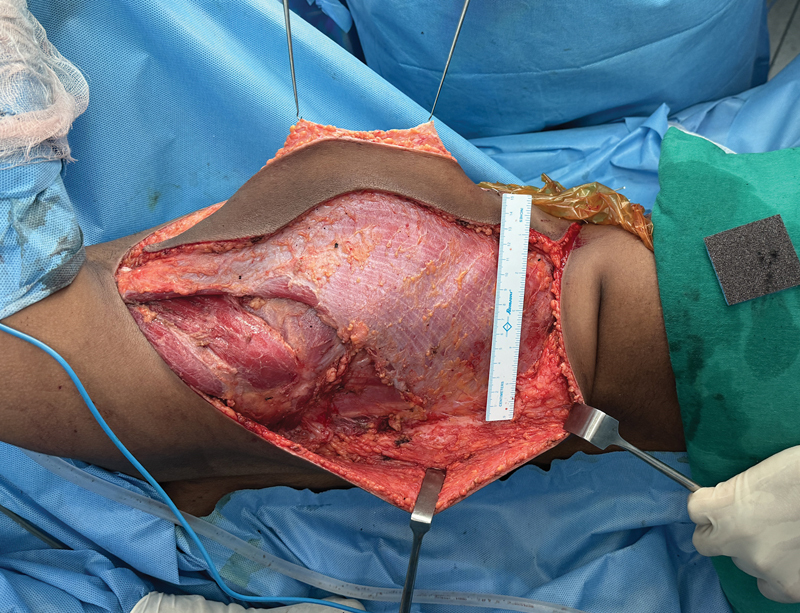
Flap post-elevation.

**Fig. 4:**
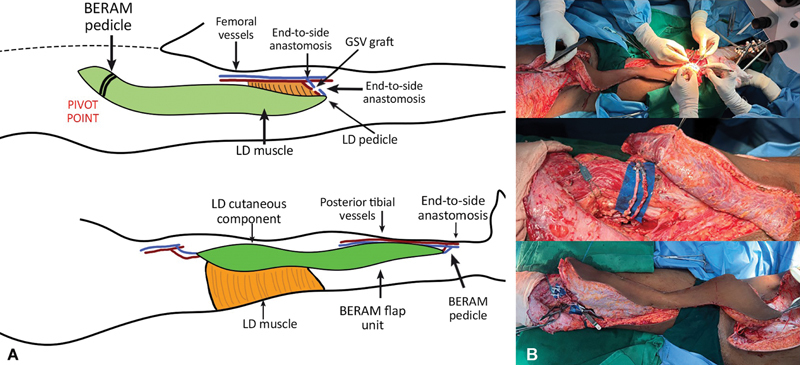
( **A** ) Schematic diagram showing plan of waltzing and anastomosis of thoracodorsal and deep inferior epigastric pedicle. ( **B** ) Detaching one end of the flap, that is, latissimus dorsi (LD) flap from the source and waltzing, pivoting on the deep inferior epigastric pedicle down to cover the knee joint with simultaneous donor site closure, anastomosis of thoracodorsal pedicle to femoral vessels in end-to-side fashion using interposition great saphenous vein graft harvested from the left side and just before division of the DIEA pedicle showing the length of the fully extended flap.

**Fig. 5:**
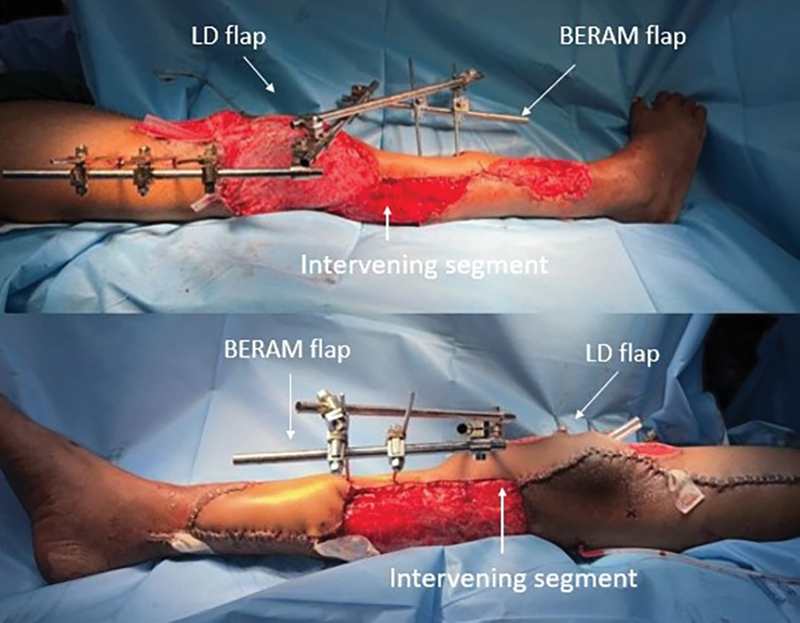
Flap post-inset on table. Latissimus dorsi (LD) muscle covered the knee joint circumferentially and boomerang-shaped extended rectus abdominis myocutaneous (BERAM) portion covered the exposed tibia.

**Fig. 6:**
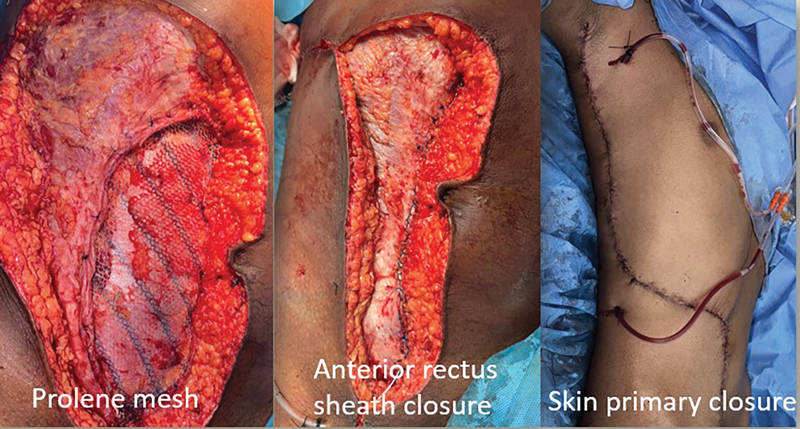
Donor site closure with Prolene mesh, anterior rectus sheath closure, and primary skin closure.

**Fig. 7:**
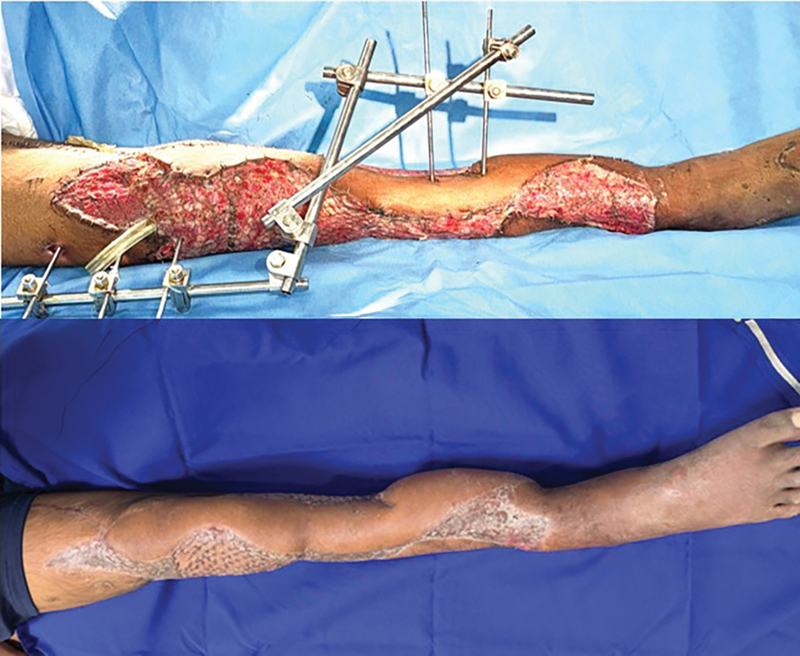
Early and late follow-up post-split-thickness skin graft (SSG).

**Fig. 8:**
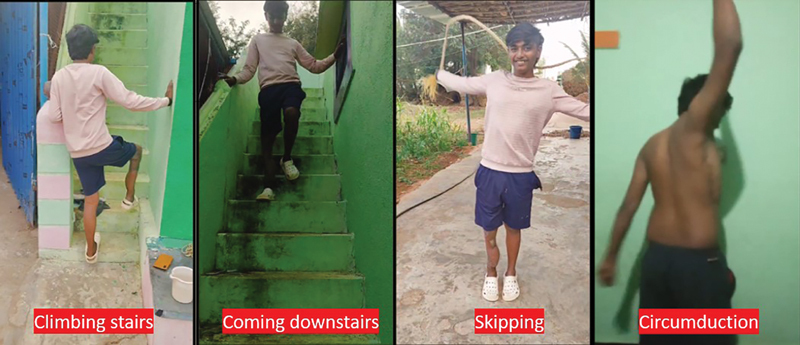
Patient functional outcome at 8 months' follow-up.

The sequence of events as to how a 7-hour surgery changed the patient's life were:

1. Wound debrided ( Fig. 1 )
2. Two-team approach was opted for, one for flap elevation and one for recipient site preparation with vein graft and vessels exposure ( Fig. 2 )
3. Great saphenous interposition vein graft harvested from the left lower limb
4. Femoral vessels exposed in Hunter's canal and PT vessels in lower one-third of the leg
5. BERAM portion of flap harvested and pedicle isolated with lateral continuation of the BERAM skin paddle onto the LD territory
6. Position changed for LD flap harvest in continuity ( Fig. 3 )
7. Flap perfusion confirmed using indocyanine green (ICG) perfusion scan ( Supplementary Fig. 2 , available in the online version) ( Video 1 )
8. Vein graft tagged and reversed for extension from the femoral vessels in an end-to-side fashion
9. LD portion disconnected and the flap waltzed down to the thigh pivoting on the deep inferior epigastric vessels, thoracodorsal vessels anastomosed to the vein grafts harvested from opposite limb in an end-to-end fashion ( Fig. 4A, B )
10. BERAM pedicle divided and waltzed down to the leg, anastomosed to PT vessels in an end-to-side fashion
11. Antibiotic beads put in the cavity on the bone and joint
12. Flap inset done over drains ( Fig. 5 )
13. Donor site closed primarily ( Fig. 6 )
14. Meshed split-thickness skin graft harvested for raw areas on postoperative day 5 ( Fig. 7 )
15. Patient discharged on day 21
16. Started weight bearing on day 90 ( Fig. 8 )

The postop outcome was uneventful barring some graft loss, which was managed by dressings.

Our lifeboat was to cover the knee joint with a free LD and a cross leg flap for covering the tibia.

Patient has foot drop at present, will be addressed once bone growth is complete.

## Discussion

A free rectus myocutaneous flap has a long pedicle with a large vascular lumen with a well-described vascular architecture of the DIEA and the paraumbilical perforators. 5 6 7 8 Because of its boomerang-like shape, Koul et al named free rectus abdominis flap design the free BERAM flap. 1

According to Taylor et al, the paraumbilical cutaneous perforator(s) can be included in the flap to create a diagonally positioned upper skin island that extends beyond the rectus muscle from the umbilicus to the costal margin. These perforators connect to the anterior branches of the lateral intercostal vessels at a 45-degree angle to the anterior axillary line via choke vessels. This idea served as the foundation for both our flap and the original BERAM flap. 8

In adults, BERAM flaps consistently yield flap lengths that are 42.2% longer than regular ALT flaps and 32.6% longer than standard LD flaps. In children, the disparity is more noticeable. 1 If the BERAM flap is raised to the mid-axillary line and the LD flap is raised with skin paddle fully centered on the muscle, the vascularity of the flap is trustworthy.

Waltzing of the flap ensures practically no cold ischemia time.

Additionally, vein grafts extended reach of the flap to the working area of the defect. The ICG perfusion analysis demonstrated good flap perfusion, particularly in the intervening tricky zone between the LD and BERAM region. 1 A high success rate, preservation of existing vessels in an injured extremity, increased operational planning flexibility, and technical simplicity for vessel access are the benefits of end-to-side anastomoses. 9 Compared to any double free flap, this flap, in our experience, offers the most tissue coverage, especially in longer than larger defects. It also saves the only available thigh for skin graft harvesting, which would otherwise be used for ALT elevation that in turn would need a skin graft for donor site coverage.

## Conclusion




**Video 1**


Extended BERAM with myocutaneous LD provides the longest tissue for coverage with a dependable pedicle for limb salvage procedures, making it a great and safe choice for extensive defects that are longer than wide enough to justify primary closure of the donor site.
