# Supplementary material for: A Game Changing, One of Its Kind Flap: The Boomerang-Shaped Extended Rectus Abdominis Myocutaneous Flap with Latissimus Dorsi Myocutaneous Flap
Source: Indian J Plast Surg. 2025 Aug 8;59(1):59–63. doi: 10.1055/s-0045-1808096 (PMC13016847; doi:10.1055/s-0045-1808096)
Supplement: Supplementary file 2 — Supplementary Material [file 10-1055-s-0045-1808096_s2523357.pdf]

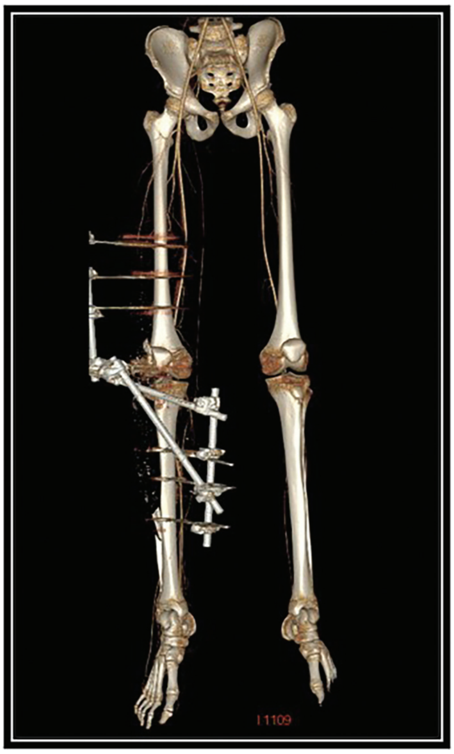

Supplementary Fig. S1 Computed tomography (CT) angio image of the patient showing single vessel status (only posterior tibial artery patent).

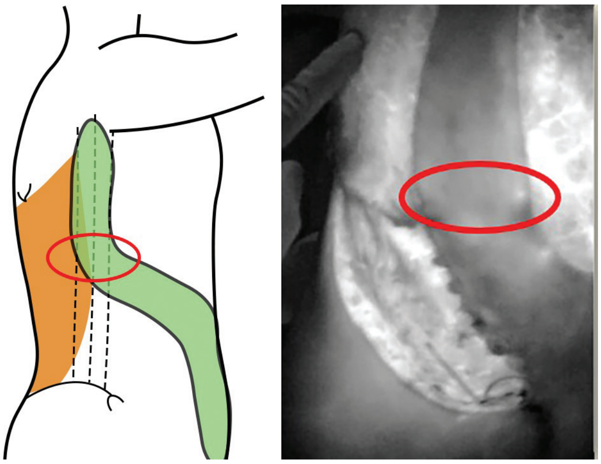

Supplementary Fig. S2 Indocyanine green (ICG) study showing good perfusion of the extended part of boomerang-shaped extended rectus abdominis myocutaneous (BERAM) flap (intervening choke zone between the two flap territories), correlation with the flap marking.

 © 2025. The Author(s).
